# President’s Message for IAGE Journal

**DOI:** 10.4103/0974-1216.71607

**Published:** 2009

**Authors:** Rakesh Sinha

**Affiliations:** Editor, JGES, 674, 16^th^ Cross road, Behind Khar Gymkhana, Khar Pali, Khar (W), Mumbai-400 052, India E-mail: beamsindia@gmail.com

**Figure:**
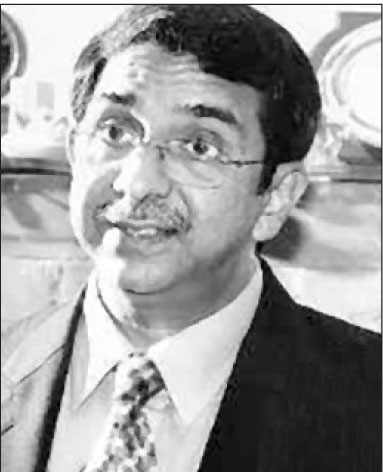


Dear Colleagues,

I am deeply honored to be the president of the Indian Association of Gynaecological Endoscopists. I take this opportunity to thank the committee members and the IAGE members and look forward to our teamwork in creating guidelines to achieve the best standard of care.

The official journal of the IAGE was released in May 2009. As a member for many years, I have witnessed the Society’s steady growth, thanks to the efforts of all its previous leaders and highly enthusiastic members. I would like to express my recognition for the excellent work and tireless efforts of our predecessors.

The acquisition of laparoscopic skills requires formal training, dedication, commitment and constant practice. In our endeavor to shorten the learning curve, we overlook the safety in laparoscopic surgery. The theme of IAGE is “Safe Endoscopy” which essentially means to improve the safety of surgical care all over the country by ensuring adherence to proven standards of care in all the laparoscopy centers.

Surgical care has been an essential component of health care worldwide. WHO has undertaken a number of global and regional initiatives to address surgical safety. We propose to create such a safety checklist for all endoscopic procedures, which can be implemented by everyone. We also look forward to a lot of workshops aiming at our theme of Safe Endoscopy. We would like to increase our membership and would love to have suggestions from all of you.

The IAGE journal is aimed to spread knowledge and update the members with the latest technology that is available. We would like to encourage our young endoscopic surgeons to contribute and share their experience.

Your membership and interest in our Society is what makes IAGE such a strong and vibrant organization. Welcome to be our members and contribute to the journal!

